# Planar Polarity Specification through Asymmetric Subcellular Localization of Fat and Dachsous

**DOI:** 10.1016/j.cub.2012.03.053

**Published:** 2012-05-22

**Authors:** Amy Brittle, Chloe Thomas, David Strutt

**Affiliations:** 1 MRC Centre for Developmental and Biomedical Genetics, University of Sheffield, Western Bank, Sheffield S10 2TN, UK; 2 Department of Biomedical Science, University of Sheffield, Western Bank, Sheffield S10 2TN, UK

## Abstract

Two pathways regulate planar polarity: the core proteins and the Fat-Dachsous-Four-jointed (Ft-Ds-Fj) system. Morphogens specify complementary expression patterns of Ds and Fj that potentially act as polarizing cues. It has been suggested that Ft-Ds-Fj-mediated cues are weak and that the core proteins amplify them [1, 2]. Another view is that the two pathways act independently to generate and propagate polarity [3, 4]: if correct, this raises the question of how gradients of Ft and Ds expression or activity might be interpreted to provide strong cellular polarizing cues and how such cues are propagated from cell to cell. Here, we demonstrate that the complementary expression of Ds and Fj results in biased Ft and Ds protein distribution across cells, with Ft and Ds accumulating on opposite edges. Furthermore, boundaries of Ft and Ds expression result in subcellular asymmetries in protein distribution that are transmitted to neighboring cells, and asymmetric Ds localization results in a corresponding asymmetric distribution of the myosin Dachs. We show that the generation of subcellular asymmetries of Ft and Ds and the core proteins is largely independent in the wing disc and additionally that ommatidial polarity in the eye can be determined without input from the Ft-Ds-Fj system, consistent with the two pathways acting in parallel.

## Highlights

► Ft and Ds become asymmetrically localized during planar polarity specification ► Ft and Ds asymmetry can be propagated from cell to cell ► Ft and Ds asymmetry promotes Dachs asymmetry and regulates polarity in the proximal wing ► Ft and Ds provide a partial polarity cue in the eye where they can act without Dachs

## Results

### Gradients of Ds and Fj Are Required for Dachs Asymmetry

**Figure 1:**
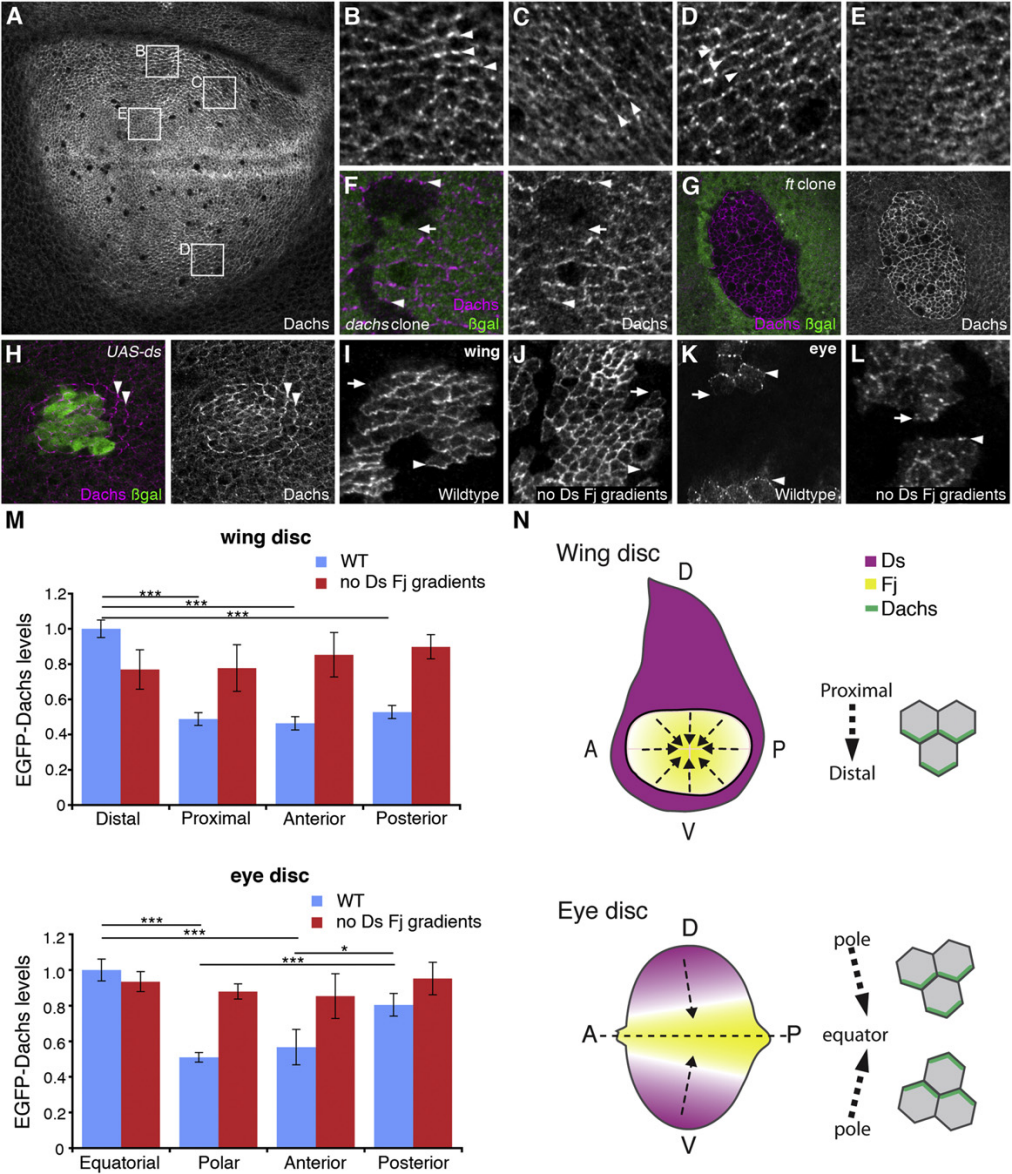
Dachs Asymmetry and Its Regulation by Fj and Ds (A–E) Confocal images of WT third-instar wing discs showing Dachs distribution revealed by immunofluorescence using an antibody raised against Dachs. (B–E) Magnifications of specific regions of the disc as marked in (A). To quantify the degree of Dachs asymmetry, we calculated the ratio of intensity of immunofluorescence on PD/AP cell junctions for the different regions, showing more on PD boundaries in regions (B), (C) and (D) (PD/AP ratios of 1.3, 1.7, and 1.24 respectively) but a negligible difference in region (E) (PD/AP ratio of 1.05). Dorsal is top and anterior left for all wing discs. (F) Wing disc containing *dachs^1^* clones near the dorsal hinge (marked by lack of β-Gal, green) labeled for Dachs (magenta). Arrowheads point to Dachs at distal junctions. Arrow points to proximal cell junctions where Dachs is reduced. (G) Wing disc containing *ft^G-rv^* clone near the hinge (marked by lack of β-Gal, green) labeled for Dachs (magenta). (H) Wing disc containing clone overexpressing Ds (*Act>stop>GAL4, UAS-lacZ/UAS-ds*, marked with β-Gal, green) labeled for Dachs (magenta). Dachs levels are increased on the boundary of the clone and 1–2 cells away (arrowheads). (I–L) EGFP-Dachs expressed in patches in the wing disc near to the dorsal hinge (I and J) and ventral eye disc (K and L). Arrowheads point to distal and arrows point to proximal cell junctions in (I) and (J). Arrowheads point to equatorial and arrows point to polar cell junctions in (K) and (L). Equator is top and anterior left. (I and K) WT (*w hsFlp; Act>stop>EGFP-dachs*). (J and L) No Ds and Fj gradients (*w hsFlp; ds^UA071^ fj^d1^ Act>stop>EGFP-dachs/ ds^38K^ fj^P1^; tub-GAL4/UAS-ds*). (M) Mean fluorescence intensity of EGFP-Dachs staining at cell junctions on the edges of clones in genotypes in (I)–(L). WT (blue bars) and no Ds and Fj gradients (red bars). Values normalized to WT levels on distal or equatorial cell junctions. Error bars show SEM. One-way analysis of variance (ANOVA) tests were applied (^∗∗∗^p < 0.001, ^∗^p < 0.05, comparisons between columns linked by bars). EGFP-Dachs is significantly higher on distal cell junctions in the wing and significantly higher on equatorial and posterior cell junctions in the eye. No statistical difference was found between levels on cell junctions in the absence of Ds and Fj gradients (red bars). (N) Ds (magenta) and Fj (yellow) expression patterns in the wing and eye disc and corresponding direction of Dachs (green) asymmetry. In the wing disc, Ds levels are high in the hinge with lower levels in the wing pouch resulting in a strong boundary of Ds expression. Fj is expressed in the center of the wing pouch with a gradient toward the edge of the pouch. Dachs asymmetry forms on the PD axis (arrows) in alignment with these Ds and Fj boundaries or gradients with high levels of Dachs on distal cell junctions. In the eye, Ds is expressed in a gradient that is high at the poles but also slightly higher anteriorly. Fj is expressed in an opposing gradient with high expression at the equator. Dachs is polarized in alignment with these gradients with high Dachs on the equatorial and posterior side of the cells. Labels refer to dorsal (D), ventral (V), anterior (A), and posterior (P). See also Figure S1.

To explore the generation of cellular asymmetry by Fat-Dachsous-Four-jointed (Ft-Ds-Fj), we generated a Dachs antibody. Using this, we see Dachs enrichment along proximodistal (PD) apicolateral cell boundaries within the third-instar wing pouch consistent with previous reports using an epitope-tagged protein (Figure 1A; [5, 6]). Dachs asymmetry is most obvious dorsally, near the edge of the wing pouch (Figures 1B and 1C) but can also be seen ventrally (Figure 1D). Asymmetry is less obvious toward the center of the wing pouch (Figure 1E). On the boundaries of *dachs* mutant clones, Dachs is seen enriched on distal cell edges (Figure 1F), as is EGFP-Dachs expressed from a transgene (Figures 1I and 1M), supporting previous findings [5, 6]. In the eye disc, Dachs is also asymmetrically localized before the furrow, primarily on equatorial cell edges but also with a posterior bias (see Figures S1B–S1E available online; Figures 1K and 1M). This is consistent with the direction of the Ds and Fj expression gradients (Figure S1A; Figure 1N [7, 8]) and also of cell division orientation in the eye [9]. Thus, Dachs asymmetry in the wing and eye maintains a consistent relationship to the Ds and Fj gradients, pointing toward high Fj and away from high Ds (Figure 1N).

Dachs accumulation at apicolateral junctions is regulated by Ft, as in *ft* mutant clones Dachs concentrates strongly around cell edges, and PD asymmetry is lost ([5]; Figure 1G). We tested whether Ds is required for this junctional accumulation. However, in *ds* mutant clones, Dachs actually shows slightly increased junctional accumulation (Figure S1F), and in *ds ft* double mutant clones, Dachs concentrates in a similar manner as in *ft* mutant clones (Figure S1G).

How the Ft-Ds-Fj system might generate cellular asymmetries that regulate Dachs localization is unknown. It has been suggested that Ds and Fj expression gradients generate small differences in Ft and Ds binding across the cell axis leading to an asymmetry of Ft activity [3, 6, 10, 11], which might then be translated into a strong asymmetry of Dachs localization. Alternatively, in the wing disc, the juxtaposition of boundaries of Fj and Ds expression at the pouch-hinge boundary might polarize Dachs [12]. Furthermore, it has been proposed that boundaries of Ft and Ds expression could alter the balance of Ft and Ds binding at cell edges and that this change might be propagated for several cells [3, 5, 6].

We tested the relevance of the Ds and Fj expression patterns by examining Dachs asymmetry in clones expressing EGFP-Dachs, in a background with no Fj and uniform Ds expression. In the absence of Ds and Fj gradients or boundaries, Dachs distal asymmetry was lost in the wing (Figures 1J and 1M). Similarly, EGFP-Dachs asymmetry in the eye was lost when Ds and Fj gradients were removed (Figures 1L and 1M).

To investigate whether boundaries of Ft and Ds expression are sufficient to produce Dachs asymmetry, we generated Ds overexpression clones. In wing and eye discs, Dachs levels are increased on the boundary of clones and also increased and strongly polarized for several cells away from the boundary (Figure 1H; Figure S1H), consistent with Ft activity being altered outside the boundary of Ds overexpression and this being propagated from cell to cell.

### Ft and Ds Asymmetric Distribution

**Figure 2:**
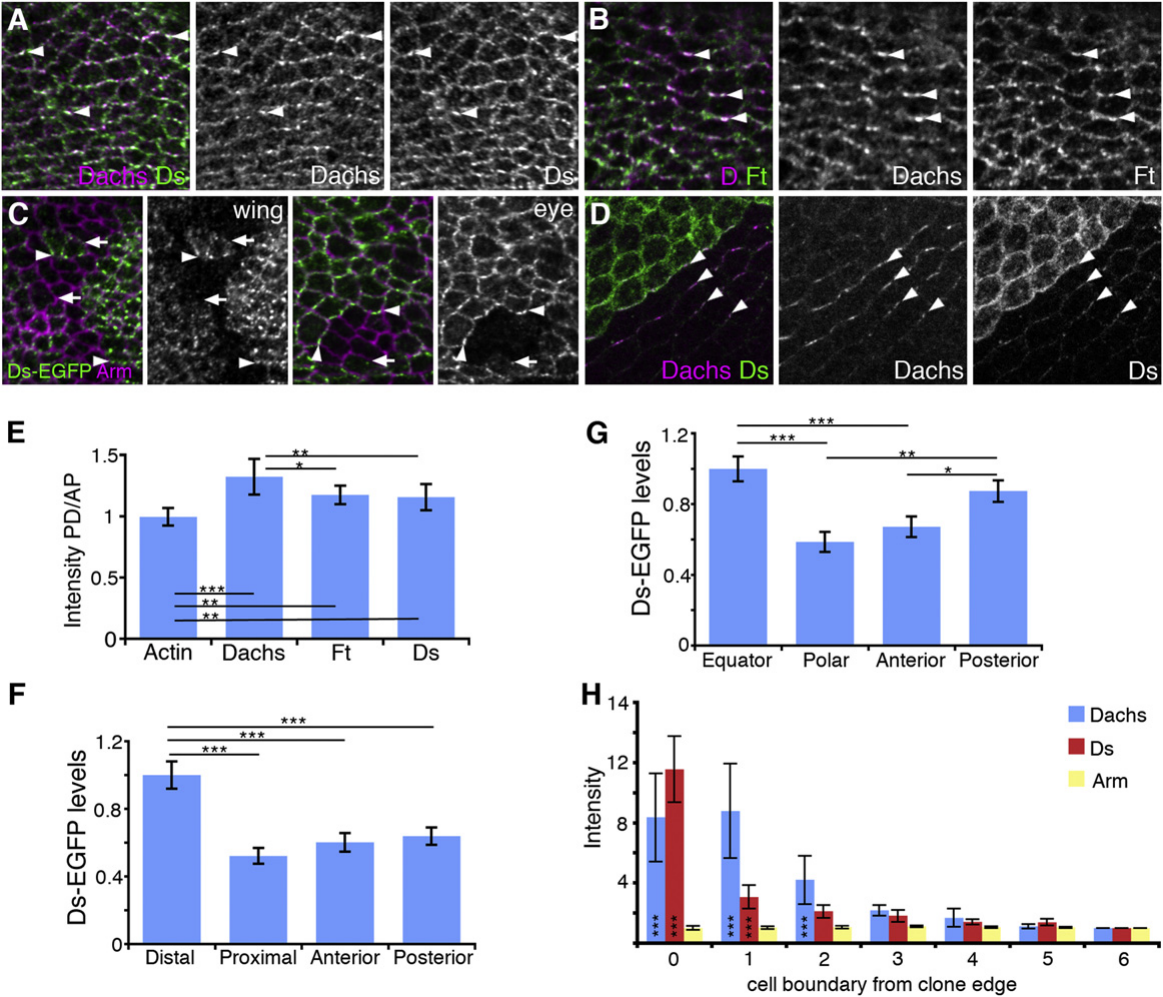
Asymmetry of Ft and Ds Distribution (A and B) Confocal images of apicolateral junctions in third-instar wing discs near the dorsal hinge. Labeled for (A) Dachs (magenta) and Ds (green) and (B) Dachs (magenta) and Ft (green). Arrowheads point to Dachs and Ds or Ft colocalization on PD cell boundaries. (C) Images of patches of Ds-EGFP expression in the wing and dorsal eye disc showing Ds-EGFP (green or white) and Arm (magenta). By using a fly strain in which *ds* is tagged at its endogenous locus with EGFP, we generated patches of Ds-EGFP expression abutting cells expressing untagged Ds but with no change in gene dosage (*ds-EGFP FRT40/ ds^+^ FRT40*). Ds-EGFP is enriched at distal cell edges in the wing disc (distal is bottom) and equatorial and posterior cell edges in the eye disc (equator is top and posterior right). Arrowheads point to Ds enrichment and arrows point to reduced Ds. (D) Twenty-eight hr pupal wing containing a clone overexpressing Ds (*Act>stop>GAL4/UAS-ds*) labeled for Ds (green) and Dachs (magenta). Arrowheads point to cell boundaries outside the overexpression clone where Dachs and Ds colocalize. Note that as Dachs and Ds asymmetry is lost by this stage of development ([2]; data not shown), the Dachs and Ds asymmetry generated around the clone is due to Ds overexpression. Additionally asymmetric localization is more easily observed in the pupal wing, because cells are larger and more uniform than in wing and eye discs and cell division is no longer occurring. (E) Ratio of mean fluorescence intensity of Actin, Dachs, Ft, and Ds labeling on PD compared to AP cell boundaries in wing discs close to the dorsal hinge. Error bars show SEM between wing discs (n = 10). A one-way ANOVA test was applied (significance levels [^∗∗∗^p < 0.001, ^∗∗^p < 0.01, ^∗^p < 0.05] between columns linked by bars). Dachs, Ds, and Ft show enrichment on PD boundaries compared to cortical actin. Enrichment of Ft (^∗^p > 0.05) and Ds (^∗∗^p < 0.01) is not as strong as Dachs. (F and G) Mean fluorescence intensity levels of Ds-EGFP staining at cell junctions on the edges of clones in wing (F) and eye discs (G) normalized to WT levels on distal or equatorial cell junctions. Error bars show SEM. One-way ANOVA tests were applied (significant differences linked by bars). In wing discs, Ds-EGFP is significantly higher on distal cell junctions than posterior, anterior, or proximal junctions. In the eye, Ds-EGFP is signficantly higher on equatorial and posterior cell junctions than polar or anterior boundaries. (H) Mean fluorescence intensity of Dachs (blue), Ds (red), and Arm (yellow) on cell boundaries parallel to *UAS-ds* overexpression clones in the pupal wing. Intensity was normalized to mean levels on boundaries away from the clone. The cell boundary at the clone edge is labeled 0 (zero). Error bars show SEM between clones (n = 10). A one-way ANOVA test was applied comparing each column with values on cell boundaries away from the clone (column 6). When averaged over ten clones, significant increases in Dachs and Ds were only found around 1–2 cells away from the clone boundary, although in some clones, differences are visible by eye 3–4 cells away; for example, see the clone in (D). See also Figure S2.

The failure to observe visible asymmetries in Ft and Ds distributions [2, 8, 13] has led to the contention that any differences in Ft and Ds activity across the axis of each cell would be only small [2, 3, 11]. To reassess this, we examined Ds and Ft protein localization in areas of the wing pouch that show strong Dachs asymmetry. Ft and Ds colocalize with strong puncta of Dachs staining, although there is a proportion of Ft and Ds that does not localize with Dachs (Figures 2A and 2B). To quantify distribution, we measured the mean intensity of Dachs, Ft, and Ds on PD and anteroposterior (AP) cell boundaries. As expected, Dachs is enriched on PD boundaries as compared to uniformly distributed cortical actin (Figure 2E). Notably, Ft and Ds immunolabeling also shows a concentration on PD compared to AP boundaries (Figure 2E). These data suggest that asymmetry of Ft and Ds distribution, rather than simply Ft activity, leads to Dachs asymmetry. Unexpectedly, increased Ft and Ds asymmetry is seen in *dachs* mutant discs (Figures S2A–S2F), indicating that the mechanism for generation of Ft and Ds asymmetry does not rely on Dachs and in fact that Dachs activity opposes it.

Because Ft and Ds bind heterophilically [10], we would predict that Ft and Ds should be distributed to opposite cell edges. To examine this, we generated a fly strain in which the *EGFP* coding sequence is inserted at the end of the *ds* coding sequence in the endogenous *ds* locus. This allows generation of mosaic patches of tissue in which cells expressing Ds-EGFP are juxtaposed to cells expressing Ds but in which there is no change in *ds* gene dosage between adjacent cells (which might locally alter cell polarity). Using this, we find strong enrichment of Ds-EGFP on distal cell edges in the wing disc (Figures 2C and 2F) and equatorial and posterior edges in the eye disc (Figures 2C and 2G), with relative levels on cell boundaries closely mimicking those seen for EGFP-Dachs (Figure 1M).

We also examined Ft distribution, using a transgene insertion that is thought to express Ft-EGFP under the complete endogenous *ft* promoter-enhancer regions. Due to the design of the experiment, *ft* activity levels are not constant between all cells, and this may affect the relative degree of asymmetry observed. Nevertheless, we detected enrichment of Ft-EGFP on proximal cell edges in the wing disc (Figures S2I and S2J).

Because Ft and Ds show subcellular asymmetric distributions, similar to those of Dachs, we asked whether such asymmetry could also be propagated from cell to cell. We measured Dachs and Ds levels on cell boundaries parallel to Ds overexpression clones. Dachs levels are increased up to four cell boundaries away from the clone edge, and smaller changes in Ds can also be detected colocalizing with Dachs on these boundaries (Figures 2D and 2H). This propagation of Dachs and Ds asymmetry supports a model in which Ft and Ds physically couple polarity between cells through asymmetric distribution of Ft and Ds protein.

In the wing disc, there is a sharp Ds boundary at the pouch-hinge junction and also graded Fj expression within the pouch (Figure 1N; [7, 13–15]). Because strong overexpression of Ds in clones is capable of reorienting Ds and Dachs asymmetry (Figure 2D), we investigated whether the Ds expression boundary in the wing disc is sufficient to produce the observed Dachs asymmetry by examining it in *fj* mutant wing discs in which only the Ds boundary will remain. We detected only very weak Dachs asymmetry (Figures S2G and S2H), indicating that the Ds boundary alone does not account for the observed asymmetry and that Fj activity is also required.

An implication of our observations is that Dachs accumulates at cell junctions where Ft levels are low and Ds levels are high. Loss of Ft activity leads to increased Dachs at junctions (Figure 1G; [5]), independently of Ds activity (Figure S1G). However, Dachs does not accumulate on anterior and posterior cell boundaries in the wing disc where Ft (and Ds) levels are low but only on distal boundaries, where Ft is low and Ds is high (compare Figure 1M, Figure S2J, and Figure 2D). Dachs also does not accumulate on junctions with low levels of Ft around Ft overexpression clones (Figure S2K). Taken together, this suggests a model in which Ft acts to modulate overall junctional levels of Dachs, and Ds recruits this pool of Dachs to particular junctional subdomains.

We tested whether Ds could recruit Dachs to the site of Ds-Ft binding in tissue culture cell aggregation assays (Figures S2L and S2M). Dachs was not obviously recruited by Ds; however, additional factors required for recruitment may be missing from the cell line used. We propose a role for Ds in concentrating Dachs at particular sites, perhaps through an indirect physical interaction.

### Ft-Ds-Dachs and Core Protein Asymmetry Can Be Independently Generated

**Figure 3:**
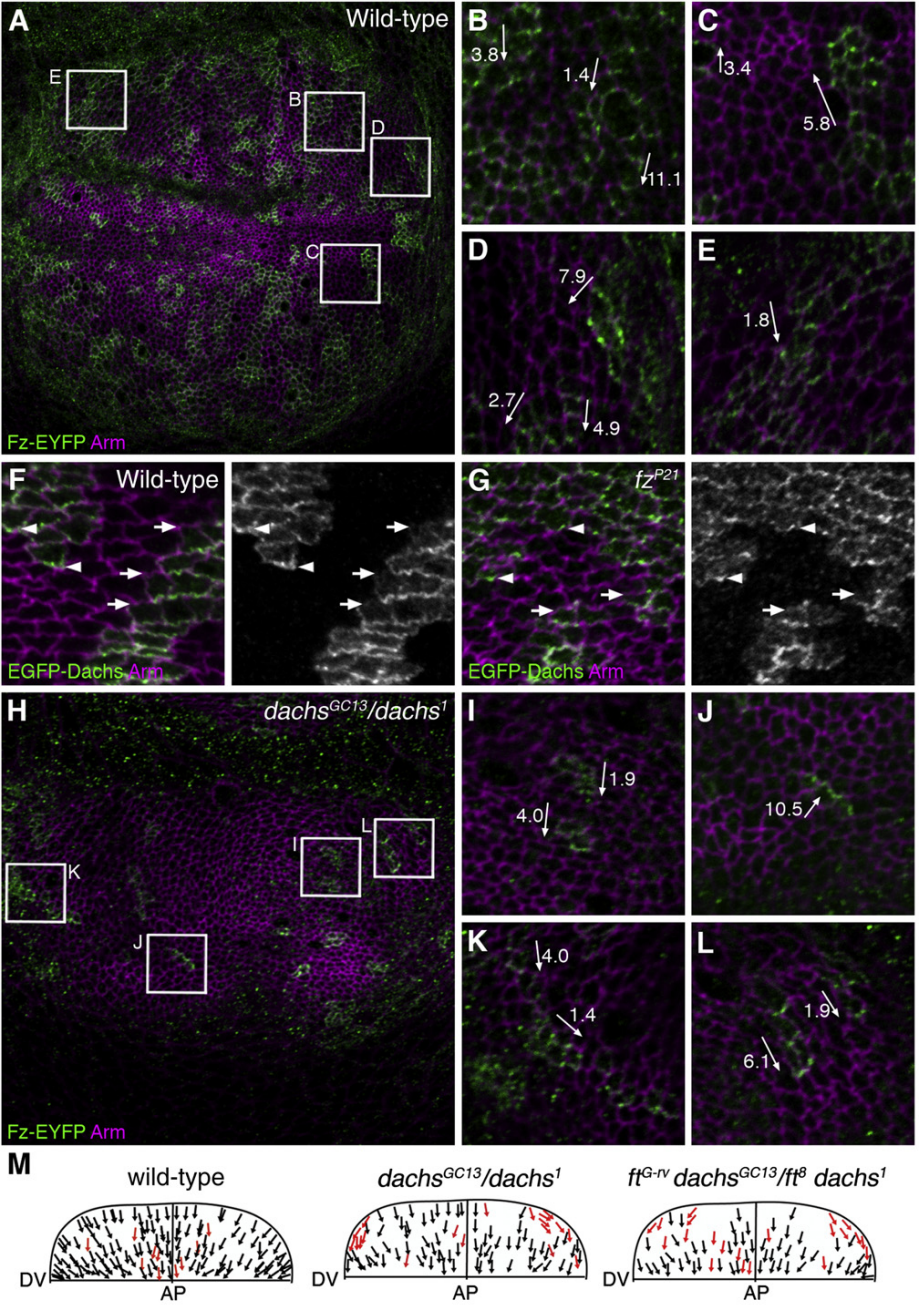
Relationship of Ft-Ds-Dachs and Core Protein Polarization (A–E) Images of EGFP-Dachs expressed in patches under the actin promoter in the wing disc near to the dorsal hinge in WT (F) and *fz^P21^* mutant background (G) labeled for GFP (green) and Arm (magenta). (B–E) Shown are magnifications of specific regions of the disc as marked in (A). Arrows show the direction of Fz-EYFP asymmetry pointing distally (toward the center of the wing pouch). Numbers refer to strength of Fz-EYFP asymmetry in individual clones as a ratio of fluorescence levels on cell boundaries on distal edges of clones versus proximal edges. There is a high degree of variation in the strength of asymmetry between clones, with an average asymmetry of 6.0 ± 0.96 (SEM). (F and G) Images of EGFP-Dachs expressed in patches under the actin promoter in the wing disc near to the dorsal hinge in WT (F) and *fz^P21^* mutant background (G) labeled for GFP (green) and Arm (magenta). Arrowheads point to distal cell junctions where EGFP-Dachs is enriched and arrows point to proximal junctions with less Dachs. Asymmetry of EGFP-Dachs was measured and no significant difference detected between WT and *fz* mutant (ratio of distal to proximal EGFP-Dachs WT = 2.9 ± 0.35 [SEM] and *fz^P21^* = 2.5 ± 0.21 [SEM], >40 clones). (H–L) Images of Fz-EYFP expressed in patches under the actin promoter in wing discs in a *dachs^GC13^/dachs^1^* mutant background labeled for GFP (green) and Arm (magenta). (I–L) Shown are magnifications of specific regions of the disc as marked in (H). In *dachs^GC13^/dachs^1^* discs, Fz-EYFP predominately points distally except in dorsal clones close to the hinge and distant from the AP boundary (L) where the direction of Fz-EYFP asymmetry follows the pouch-hinge boundary. This was not the case in WT (E). In (I)–(K), numbers refer to strength of Fz-EYFP asymmetry in individual clones as ratio of distal/proximal fluorescence levels. In (L), the ratio of Fz-EYFP fluorescence relative to the axis of the pouch-hinge boundary is indicated: PD asymmetry is negligible in these clones (PD ratios are 1.2 and 0.7). (M) Mapped orientation of Fz-EYFP asymmetry in the dorsal half of wing discs in WT, *dachs^GC13^/dachs^1^* and *ft^G-rv^ dachs^GC13^/ ft^8^ dachs^1^*. Red arrows indicate Fz-EYFP clones in which asymmetry was misoriented (pointing away from the AP boundary). See also Figure S3.

It has previously been suggested that Ft and Ds provide polarizing cues to the core proteins [1, 2, 8], but it is also possible that asymmetric core protein localization could regulate the observed asymmetry of Ft-Ds-Dachs. Because the Ds and Fj expression patterns lead to visible asymmetry of Ft-Ds-Dachs in third-instar wing discs, we examined core protein asymmetry at this stage and found that they are also asymmetrically localized on PD boundaries (Figures 3A–3E; see also [16]) similar to the pattern of Dachs localization. We tested whether core protein activity is required for Dachs asymmetry but found that EGFP-Dachs distal localization was still present in a *fz* null mutant (Figures 3F and 3G). Furthermore, asymmetry of endogenous Dachs and Ft was still detected in *fz* mutant wing discs (Figures S3A and S3B).

Current data suggest that Ft and Ds are largely dispensable for polarizing the core proteins in the wing: except for weak defects in proximal regions, trichome polarity is normal when Ds and Fj gradients are removed [10, 17], in *ft* mutants rescued by overexpression of a form of Ft lacking its extracellular domain [18], or by reduction of Warts-Hippo (Wts-Hpo) pathway activity [5, 19], which suppresses the overgrowth phenotype associated with loss of Ft and Ds signaling (reviewed in [20, 21]). Consistent with these findings, in *ds dachs* adult wings we also observe a suppression of overgrowth and only weak proximal polarity defects (Figures S3H–S3K).

We examined whether Ft-Ds-Dachs polarity in the third-instar wing disc is required for the generation of core protein asymmetry. Frizzled-EYFP (Fz-EYFP) distal asymmetry was maintained in both *dachs* and *ft dachs* mutant wing discs (Figures 3H–3L; Figures S3C–S3G), and the strength of asymmetry was not significantly altered (ratio of distal to proximal Fz-EYFP in wild-type [WT] = 6.0 ± 0.96 [SEM], *dachs* mutant = 4.6 ± 0.83, *ft dachs* mutant = 5.0 ± 0.37). However, by mapping the orientation of Fz-EYFP in clones in the dorsal half of the wing disc, we detected a change in Fz-EYFP orientation close to the pouch-hinge boundary (which will later become the proximal wing) in both mutant backgrounds, such that it no longer points distally but follows the pouch-hinge boundary (Figure 3M). Because *dachs* and *ft dachs* mutants share a similar phenotype, the defect in Fz-EYFP localization is likely due to the lack of Dachs.

We conclude that in most of the wing, Ft and Ds polarity does not play an essential role in polarizing the core, implying that additional unknown cues exist. However, Ft and Ds regulation of Dachs is required to ensure that the core proteins are correctly polarized in the proximal wing as early as the third-instar stage of development. Consistent with this, some disruption of core protein asymmetry is seen in *dachs* mutant pupal wings [22], and in the adult, planar polarity defects are weak and restricted to proximal regions (Figure S3K; [5, 22]). Dachs may provide a direct cue to the core in the proximal wing or be required permissively for other unknown cues to act.

### The Core System Receives Ft-Ds-Fj-Dependent and -Independent Cues in the Eye

**Figure 4:**
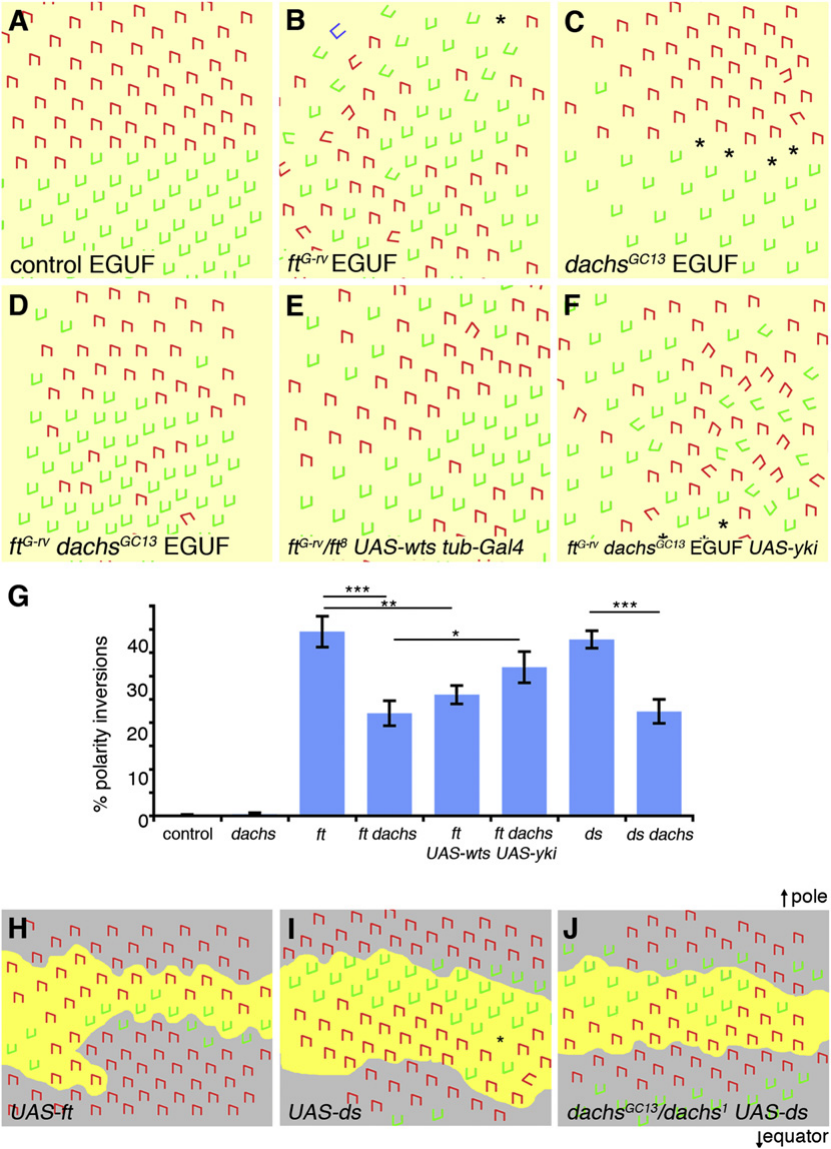
Regulation of Ommatidial Polarity by the Ft-Ds Pathway and Dachs (A–F) Diagrams representing the chirality and orientation of ommatidia in the adult eye. Dorsal chiral form of ommatida (red), ventral chiral form (green), achiral ommatidia (blue), and disrupted ommatidia (^∗^) marked. Eyes wholly mutant for specific genotypes were generated using the *eyeless-GAL4 UAS-FLP* (“EGUF”) system except in (E). (A) *FRT40.* (B) *ft^G-rv^ FRT40.* (C) *dachs^GC13^ FRT40.* (D) *ft^G-rv^ dachs^GC13^FRT40.* (E) *ft^G-rv^ dachs^GC13^ UAS-wts / ft^8^ dachs^1^ tub-GAL4.* (F) *ft^G-rv^ dachs^GC13^ FRT40 UAS-yki.* (G) Quantification of polarity defects. Columns 1–6 are the same genotypes as in (A)–(F). Additional genotypes are *ds^UA071^ /ds^38k^* (column 7) and *ds^UA071^ d^GC13^ / ds^38k^ d^1^* (column 8). Error bars show SEM between eyes (n > 6). A one-way ANOVA test was applied (^∗∗∗^p < 0.001, ^∗∗^p < 0.01, ^∗^p < 0.05 comparisons between columns linked by bars). *ft^G-rv^/ ft^8^* eyes could not be examined for comparison with genotype in (E) because the combination is lethal. However, *ft^8^* EGUF eyes have a level of inversions (data not shown) similar to *ft^G-rv^* eyes, suggesting that the polarity phenotype is suppressed in (E). (H–J) Diagrams representing orientation of ommatidia around overexpression clones (yellow). (H) *Act≫GAL4/UAS-ft.* (I) *Act≫GAL4/UAS-ds.* (J) *dachs^GC13^/dachs^1^ Act≫GAL4 UAS-ds.* Equator is toward the bottom and pole toward the top of the images. Nonautonomous inversions in polarity occur on the equatorial side of *UAS-ft* clones (H) and on the polar side of *UAS-ds* clones (I). *UAS-ds* clones in a *dachs* mutant (J) produce polar inversions at the same level with an average of number of inversions per clone in WT of 2.8 (n = 4) and 2.7 in *dachs* (n = 7). See also Figure S4.

Although Ft-Ds-Fj are only essential for polarization of the core proteins in the proximal wing, Ds and Fj gradients are required throughout the eye [8, 17], suggesting that here Ft and Ds asymmetry may play a more general role in polarization of the core proteins and thus determination of ommatidial polarity. To investigate, we suppressed Yorkie (Yki) activity, the transcriptional activator of the Wts-Hpo pathway, in *ft* or *ds* mutant eyes, by either removing Dachs or overexpressing Wts, and we found that both growth defects (data not shown) and the planar polarity phenotype were suppressed (Figures 4A–4E, and 4G), although some ommatidial polarity defects were still evident. This suggests that high Yki activity contributes to the strong planar polarity phenotype associated with *ft* and *ds* mutants, as has been shown in the wing [19]. Consistent with this, altering Wts-Hpo activity in the eye can produce planar polarity phenotypes (Figures S4A–S4F). Furthermore, overexpression of Yki in *ft dachs* eyes increased polarity defects (Figures 4F and 4G).

Hence, as in the wing, eye tissue is able to at least partly polarize in the absence of *ft* and *ds* activity, as long as the Wts-Hpo pathway is suppressed. Thus, one or more inputs other than Ft and Ds must supply directional information to the core proteins. However, unlike in the wing, where polarity phenotypes are completely suppressed away from proximal regions, there are residual polarity defects throughout *ft dachs* and *ds dachs* eyes, suggesting that Ft and Ds provide patterning input throughout the tissue. This input appears not to be mediated by Dachs because *dachs* mutant eyes have significantly milder ommatidial polarity phenotypes than *ft dachs* mutants (Figures 4C and 4G).

To further confirm the ability of Ft and Ds to provide polarity cues in the eye independently of Dachs activity, we looked at the effects of Ft and Ds overexpression clones. Notably, in the eye, Ft overexpression causes changes in polarity in WT tissue on the equatorial side of clones (Figure 4H) and Ds overexpression causes reorientation of polarity on the polar side (Figure 4I)—these being the opposite effects to loss-of-function clones for either gene [8, 23]. However, clones overexpressing Ds in a *dachs* mutant background show a similar level of nonautonomous polarity inversions as those in a WT background (Figure 4J). Thus, we conclude that Ft and Ds use a Dachs-independent mechanism to influence core-mediated polarity in the eye.

## Discussion

Our results demonstrate the importance of gradients and boundaries of Ds and Fj expression in the generation of cellular asymmetry. Previous reports have suggested that weak differences in Ft and Ds binding across cells could be amplified to produce asymmetric localization of downstream pathway effectors such as Dachs [6]. Here, we report significant asymmetry of both Ft and Ds localization, suggesting that physical polarization of these proteins is an important part of the mechanism by which Ft-Ds-Fj generate polarity. We thus reveal the Ft-Ds-Fj system as a mechanism for converting long-range morphogen-induced gene expression patterns into planar polarity cues at the level of individual cells.

In the wing disc, Dachs asymmetry is particularly prominent at the pouch-hinge boundary where a strong disparity in Ds levels exists. In this situation, the Ds boundary may contribute to the high level of asymmetry, for instance, via the feed-forward mechanism proposed by Zecca and Struhl [12] that suggests that Dachs asymmetry is produced by strong differences in Ds and Ft binding between neighboring cells, that is passed from cell to cell as the wing grows. However, we also detect strong asymmetry of Ds and Dachs in the eye disc, where there is no evidence for sharp disparities of Ds or Fj, consistent with expression gradients providing sufficient cues. We also see Dachs asymmetry in 6 hr pupal wings (data not shown) consistent with Ft-Ds-Fj signaling continuing to provide polarizing cues after the third-instar stage [10, 24, 25].

The ability of shallow expression gradients to produce observable asymmetry of Ft and Ds distribution is unexpected. A possible mechanism is that a weak asymmetry in activity or protein distribution across the cell is amplified by a feedback loop to produce an observable protein asymmetry, in a manner similar to that suggested for the generation of core protein asymmetry [26, 27]. Notably, Dachs does not seem to be part of any such amplification mechanism. Indeed loss of Dachs activity appears to promote Ft and Ds asymmetry. It may be that cell divisions, which are reduced in *dachs* mutants, disrupt the appearance of asymmetry, possibly explaining the high level of variance of asymmetry of Dachs, Ft and Ds in WT tissue. To understand further how the asymmetry of Ft and Ds is achieved, and whether this requires an amplification mechanism, it will be necessary to combine more detailed quantitative analyses together with computational approaches.

Our data suggest that Ft and Ds asymmetry leads directly to the observed Dachs asymmetry in both wing and eye discs. Although we failed to detect direct interactions between Ds and Dachs, the colocalization and the similar degree of subcellular asymmetry observed for these proteins support a model in which Ds recruits Dachs.

Finally, we have reassessed the link between Ft-Ds-Fj and the core planar polarity proteins. In the wing, we demonstrate that throughout much of the third-instar disc, both Ft-Ds-Dachs and the core proteins independently adopt PD-oriented subcellular localizations, most likely under the influence of the morphogen gradients that pattern the axes of the tissue (see [3] and A. Sagner and S. Eaton, personal communication). However, in the most proximal regions of the wing (adjacent to the pouch-hinge boundary in the disc), Ft-Ds-Fj appear to act via Dachs to ensure correct polarization of the core proteins. The mechanism behind Dachs regulation of the core needs further investigation, but because Dachs plays a role in orientated cell division and influences apicolateral junctional length [28], these factors may be involved.

In the eye, Ft-Ds-Fj seem to play a more general role in polarizing the core proteins throughout the tissue, apparently independently of Dachs activity. Ft-Ds-Fj may also provide a Dachs-independent input to the core in the wing, but data presented here and in previous reports suggest that it is at best redundant. Even in the eye, Ft-Ds-Fj are not absolutely essential for the core to polarize, indicating that there are other unknown inputs.

An important observation is that complete loss of *ft* or *ds* activity in the eye or wing results in very strong defects in core protein polarity [2, 8, 13], but when overgrowth is suppressed in these backgrounds via manipulation of Wts-Hpo pathway activity, then much milder defects are observed (this work and [5, 18, 19]). On one hand, excessive cell division alone may disrupt the process of planar polarity establishment by the core proteins, possibly due to asymmetric localization being lost each time a cell undergoes mitosis [29]. Alternatively, Ft-Ds-Fj-mediated polarity cues may constitute more important inputs to the core proteins in proliferating tissues. Finally, it is possible that other Wts-Hpo pathway transcriptional targets, not related to growth, contribute to the planar polarity phenotype.

Overall, our data support a model in which the Ft-Ds-Fj system and core planar polarity proteins act independently to generate and propagate planar polarity through the asymmetric subcellular distribution of their protein components. We find no evidence that the core proteins can influence the asymmetry of the Ft-Ds-Fj system; however, in particular contexts, the Ft-Ds-Fj system can act through different effectors to influence core protein polarity.

## Supplemental Information

Document S1. Figures S1–S4 and Supplemental Experimental Procedures
