# Supplementary material for: Planar Polarity Specification through Asymmetric Subcellular Localization of Fat and Dachsous
Source: Curr Biol. 2012 May 22;22(10):907–14. doi: 10.1016/j.cub.2012.03.053 (PMC3362735; doi:10.1016/j.cub.2012.03.053)
Supplement: Document S1. Figures S1–S4 and Supplemental Experimental Procedures [file mmc1.pdf]

Current Biology, Volume *22*

Supplemental Information

Planar Polarity Specification through Asymmetric Subcellular Localization of Fat and Dachsous

Amy Brittle, Chloe Thomas, and David Strutt

Supplemental Inventory

1. Supplemental Figures

Figure S1, related to Figure 1

Figure S2, related to Figure 2

Figure S3, related to Figure 3

Figure S4, related to Figure 4

2. Supplemental Experimental Procedures

3. Supplemental References

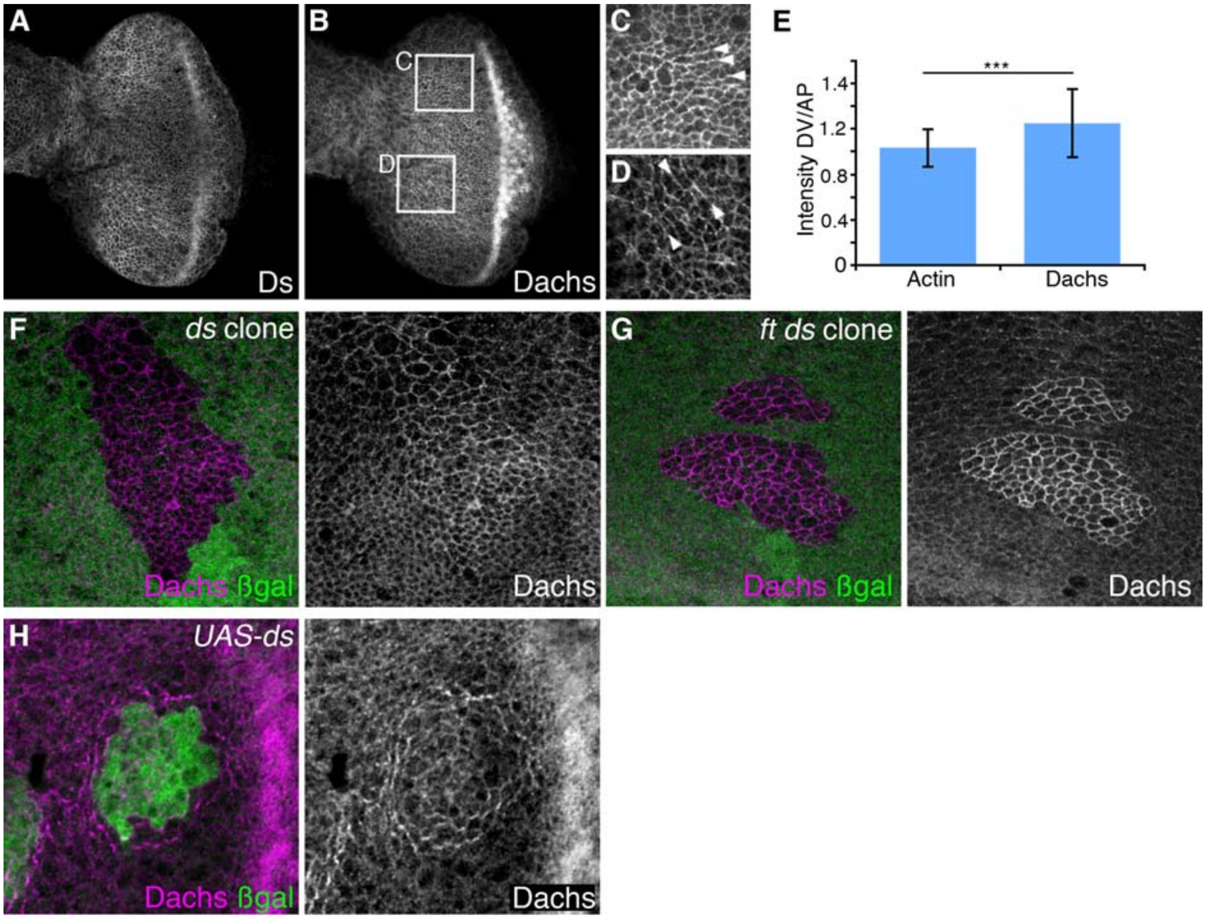

Figure S1, Related to Figure 1

(A) Third instar eye disc labelled for Ds. Ds levels are higher at the poles and also anteriorly.

(B-D) Third instar eye disc labelled for Dachs. (C,D) magnifications of specific regions of the disc as marked in B. Dachs is enriched on DV cell boundaries (arrowheads in C) but in more anterior regions of the disc closer to the equator Dachs enrichment is also biased on the AP axis (D).

(E) Quantification of Dachs levels on cell boundaries corresponding to region shown in (C). Dachs is significantly enriched on DV compared to AP boundaries compared to cortical actin (Student's t-test ***p<0.001). Error bars show SEM between wing discs.

(F) Wing disc containing *ds*^{UA071} clone near the dorsal hinge (marked by lack of ßGal, green) labelled for Dachs (magenta).

(G) Wing disc containing *ds*^{UA071} *ft*$^{G\text{-}rv}$ clones near the hinge (marked by lack of ßGal, green) labelled for Dachs (magenta).

(H) Eye disc containing clone overexpressing Ds (*Act>stop>GAL4, UAS-lacZ/UAS-ds*, marked with ßGal, green) labelled for Dachs (magenta). Dachs levels are increased on the boundary of the clone and 1-2 cells away.

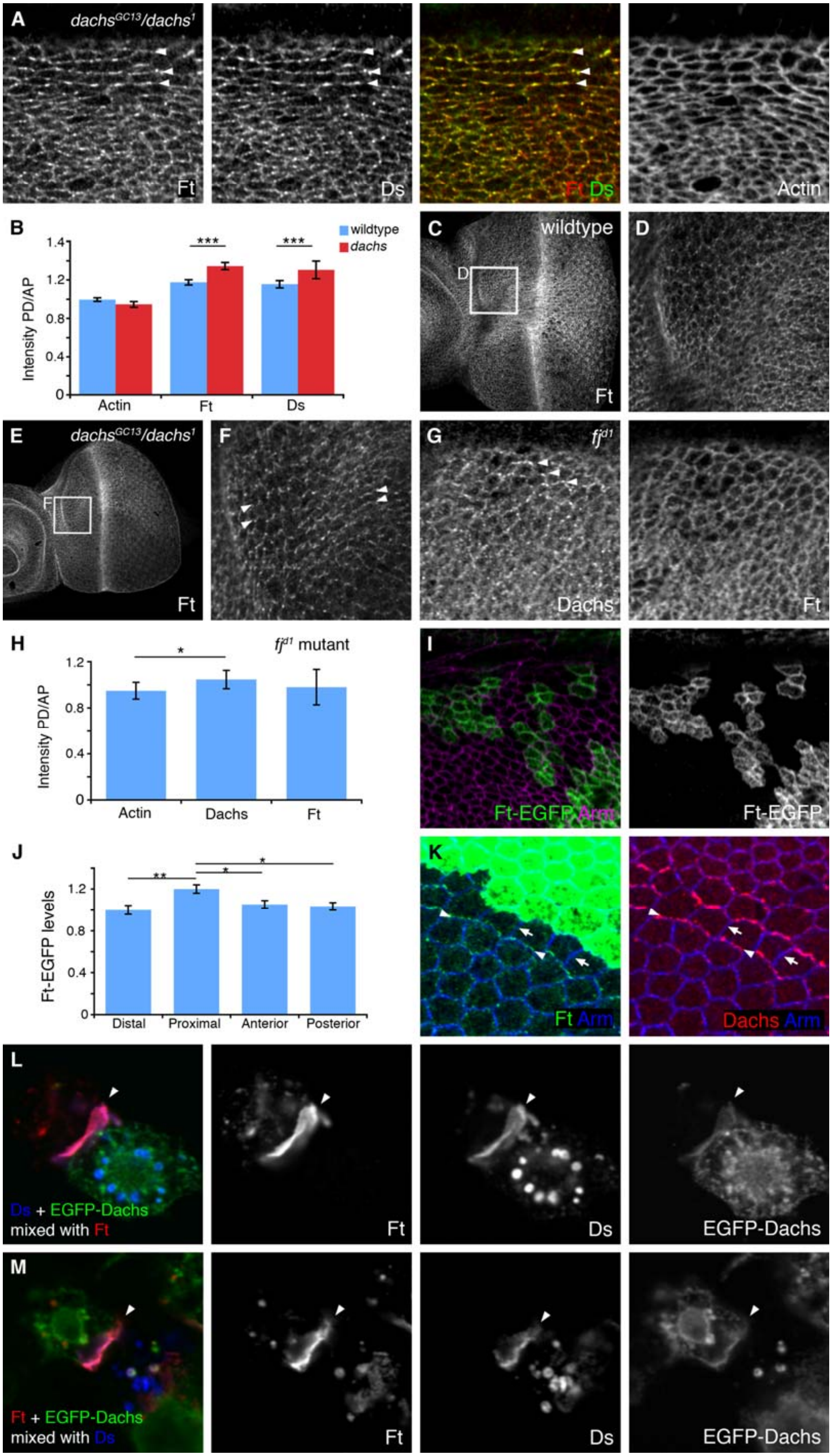

A
dachs^GC13/dachs^1
Ft
Ds
Ft Ds
Actin
B
wildtype
dachs
Intensity PD/AP
1.4
1.2
0.8
0.4
0
***
***
Actin
Ft
Ds
C
wildtype
D
Ft
D
E
dachs^GC13/dachs^1
F
Ft
F
G
fj^d1
Dachs
Ft
H
fj^d1 mutant
Intensity PD/AP
1.2
0.8
0.4
0
*
Actin
Dachs
Ft
I
Ft-EGFP Arm
Ft-EGFP
J
Ft-EGFP levels
1.2
0.8
0.4
0
**
*
*
Distal
Proximal
Anterior
Posterior
K
Ft Arm
Dachs Arm
L
Ds + EGFP-Dachs
mixed with Ft
Ft
Ds
EGFP-Dachs
M
Ft + EGFP-Dachs
mixed with Ds
Ft
Ds
EGFP-Dachs

Figure S2, Related to Figure 2

(A) Immunofluorescence confocal images of a *dachs*^{GC13}/*dachs*^{1} wing disc near the dorsal hinge labelled for Ds (white or green), Ft (white or red), actin (white).

(B) Ratio of mean fluorescence intensity of actin, Ft and Ds staining on PD compared to AP cell boundaries in wing discs close to the dorsal hinge in wildtype (blue) and *dachs*^{GC13}/*dachs*^{1} mutant (red). Error bars show SEM between wing discs (n=10). Student's t-tests were applied. Enrichment of Ds and Ft on PD boundaries in *dachs*^{GC13}/*dachs*^{1} discs was significantly greater than in wildtype ($p<0.001$).

(C,D) Wild-type third instar eye disc labelled for Ft. (D) magnification of specific region as marked in (C). Ft asymmetry is not obvious.

(E,F) *dachs*^{GC13}/*dachs*^{1} third instar eye disc labelled for Ft. (F) magnification of specific region as marked in (E). Ft enrichment at cell boundaries on the DV axis can be seen (arrowheads).

(G) Images of *fj*^{d1} wing disc near to dorsal hinge labelled for Dachs and Ft. Arrowheads indicate examples of visible Dachs asymmetry.

(H) Ratio of mean fluorescence intensity of actin, Dachs and Ft staining on PD compared to AP cell boundaries in wing discs close to the dorsal hinge in *fj*^{d1}. Error bars show SEM between wing discs. A one-way ANOVA test was applied. A small enrichment of Dachs but not Ft compared to actin on DV boundaries was detected.

(I) Ft-EGFP expression in the wing disc labelled for GFP (green) and Arm (magenta). Ft-EGFP localises to apical junctions.

(J) Mean fluorescence intensity levels of Ft-EGFP staining at cell junctions on the edges of clones in wing discs, normalised to levels on distal cell junctions. Error bars show SEM. A one-way ANOVA test was applied. By eye asymmetry of Ft-EGFP is not obvious (panel I), however, measurements reveal a modest but significant increase in Ft-EGFP levels on proximal cell junctions compared to distal, anterior or proximal junctions.

(K) *UAS-ft* overexpression clone in pupal wing labelled for Ft (green), Dachs (red) and Arm (blue). Clone marked by high level of Ft. Changes in Ft/Dachs outside the clone can be seen. Ft staining is weak on cell boundaries perpendicular to the clone (arrows) but Dachs does not accumulate here. Dachs accumulates on parallel boundaries (arrowheads) where Ds is localised (see Fig.2D).

(L,M) *Drosophila* S2 cells transfected with Ds, Ft and EGFP-Dachs expressed under the actin promoter. Labelled for Ds (blue), GFP (green) and Ft (red).

(L) Cells co-transfected with Ds and EGFP-Dachs mixed with cells transfected with Ft.

(M) Cells co-transfected with Ft and EGFP-Dachs mixed with cells transfected with Ds. EGFP-Dachs was not recruited by Ds or Ft to sites of Ds/Ft binding (arrowheads).

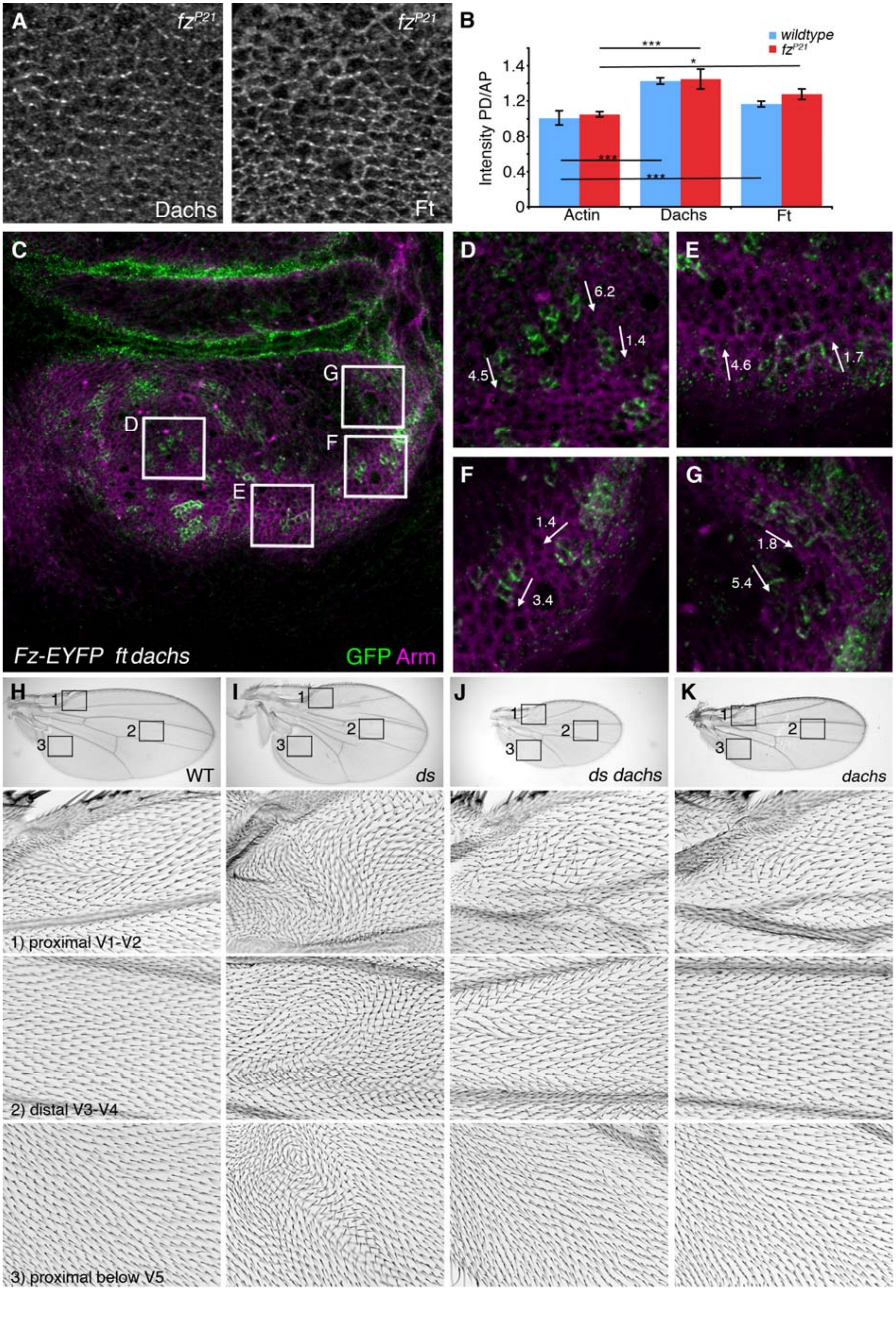

A
fz^P21
Dachs
fz^P21
Ft
B
wildtype
fz^P21
Intensity PD/AP
1.4
1.2
0.8
0.4
0
***
*
***
***
Actin
Dachs
Ft
C
G
D
F
E
Fz-EYFP ft dachs
GFP Arm
D
6.2
1.4
4.5
E
4.6
1.7
F
1.4
3.4
G
1.8
5.4
H
1
2
3
WT
I
1
2
3
ds
J
1
2
3
ds dachs
K
1
2
3
dachs
1) proximal V1-V2
2) distal V3-V4
3) proximal below V5

Figure S3, Related to Figure 3

(A) Immunofluorescence images of *fz*^{P21} wing disc near dorsal hinge labelled for Dachs and Ft to show enrichment on DV cell boundaries.

(B) Ratio of mean fluorescence intensity of actin, Dachs and Ft staining on PD compared to AP cell boundaries in wing discs close to the dorsal hinge in wildtype (blue) and *fz*^{P21} mutant (red). Error bars show SEM between wing discs. A one-way ANOVA test was applied. Enrichment of Dachs and Ft on PD boundaries in *fz*^{P21} discs was not significantly different than in wildtype.

(C-G) Images of Fz-EYFP expressed in patches under the actin promoter in wing discs in a *ft*$^{G\text{-}rv}$ *dachs*^{GC13}/ *ft*^{8} *dachs*^{1} mutant background. Labelled for GFP (green) and Arm (magenta). (D-G) show magnifications of specific regions of the disc as marked in (C). Fz-EYFP predominately points distally except in dorsal clones close to the hinge and distant from the AP boundary (G) where the direction of Fz-EYFP asymmetry follows the pouch/hinge boundary. In (D-F) numbers refer to strength of Fz-EYFP asymmetry in individual clones as ratio of distal edge to proximal edge fluorescence levels. In (G) asymmetry in the axis of the hinge/pouch boundary is indicated: PD asymmetry is negligible (1.04 and 1.08).

(H-J) Images of adult wings of indicated genotypes. (H) Wildtype, (I) *ds*^{UA071}/ *ds*^{38k}, (J) *ds*^{UA071}*d*^{GC13}/ *ds*^{38k} *d*^{1} and (K) *d*^{GC13}/ *d*^{1}. Lower panels are higher magnification images of specific regions of the wing to show hair polarity (1) proximal wing between vein 1 and 2 (V1-V2), (2) distal between veins 3 and 4 (V3-V4) and (3) proximal below vein 5 (V5). *ds*^{UA071}/ *ds*^{38k} wings (I) show strong hair swirls in all three regions but this phenotype is suppressed in *ds*^{UA071}*d*^{GC13}/ *ds*^{38k} *d*^{1} wings (J). Proximal hair swirls around veins 1 and 2 are seen in both *ds*^{UA071}*d*^{GC13}/ *ds*^{38k} *d*^{1} and *d*^{GC13}/ *d*^{1} wings. Hairs in *ds*^{UA071}*d*^{GC13}/ *ds*^{38k} *d*^{1} wings can appear slightly displaced after mounting due to unevenness of the surface of the wings. *ds dachs* mutant wings were examined, as *ft dachs* mutants were lethal.

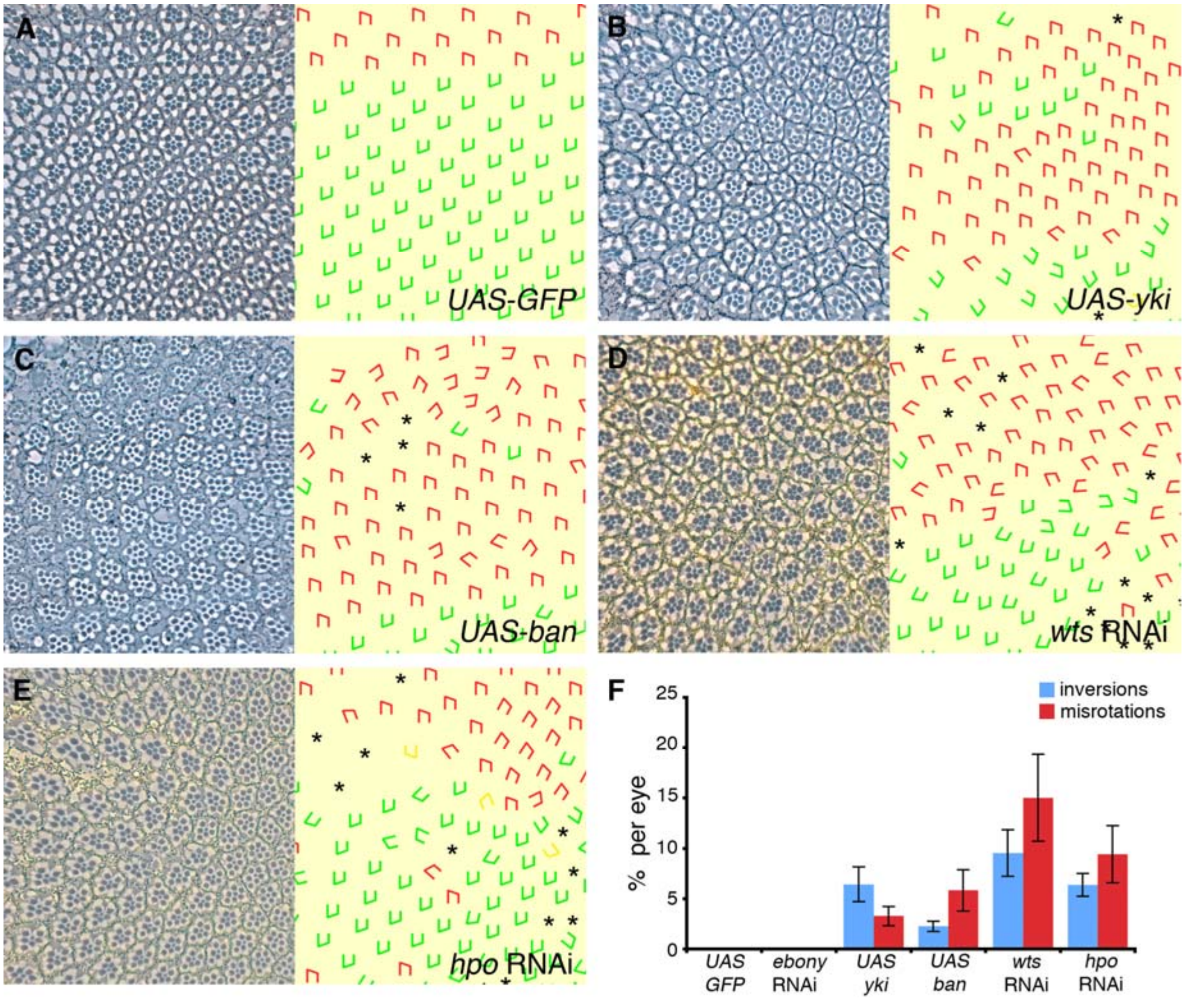

Figure S4, Related to Figure 4

(A-E) Sections of adult eye (left panels) and diagrams (right panels) representing the orientation and chirality of ommatidia. The dorsal chiral form of ommatidia (red), ventral form of ommatidia (green), wrong chirality (yellow) and disrupted ommatidia (*) are marked. *ey-GAL4* was used for overexpression or to induce RNAi at 29ºC.

(A) *UAS-GFP – UAS* overexpression control

(B) *UAS-yki* – activates transcription of Wts/Hpo pathway targets e.g *bantam, cycE, DIAP1*

(C) *UAS-bantam* – transcriptional target of Wts/Hpo pathway (a micro RNA involved in cell proliferation control)

(D) *wts RNAi* – activates Wts/Hpo pathway activity by promoting activity of the transcription factor Yki

(E) *hpo RNAi* – activates Wts/Hpo pathway activity by promoting activity of the transcription factor Yki

(F) Quantification of polarity inversions and misrotations in eyes. All manipulations of the Wts/Hpo pathway produce significant increases in inversions and misrotations compared to controls (*UAS-GFP* and *ebony* RNAi) when Student's t-test is applied. Error bars show SEM between eyes. Overexpression of *bantam*, a transcriptional target of Yki associated with overproliferation, produces polarity defects suggesting that planar polarity phenotypes caused by the Wts/Hpo pathway could be caused by excess growth. However, as the defects are mild other Yki targets could play a role. Overexpression of other targets – *cycE*, *DIAP1* and *fj* – failed to cause polarity defects (data not shown).

Supplemental Experimental Procedures

Molecular Biology

The *dachs* coding region (containing introns but lacking 5' and 3' UTRs) was cloned by PCR from pFlyFos023618 [1] and fused to EGFP to generate *pAct>stop>EGFP-dachs* and *pAc5.1-EGFP-dachs*.

A 22kb genomic fragment from BACR11D14 containing the entire coding sequence of *ft* was cloned into pUAST [2] for expression in flies.

A P[acman] construct expressing Ft-EGFP under control of the Ft genomic regulatory elements was generated from the attB-P[acman-V5:fat+] construct [3] into which the EGFP coding sequence was inserted immediately before the Stop codon of *ft* by standard recombineering methods.

Fly Strains

Alleles used ($ft^{G\text{-}rv}$, ft^{8}, ds^{UA071}, ds^{38K}, $dachs^{GC13}$, $dachs^{1}$, fj^{d1}, fj^{P1}, fz^{P21}) are described in FlyBase [4]. Clones were generated using the FLP/FRT system [5] and marked with *arm-lacZ* [6] in discs. Overexpression clones were generated using *P[w+, Act>y+>GAL4]* and marked with *P[ry+, UAS-white]* in the eye as described [7] or *UAS-lacZ*. Ds was overexpressed using *UAS-ds* ([8] or [9]).

Homologous recombination was used to target EGFP into the C-terminus of the *ds* gene at its endogenous locus using the pRK2 targeting vector [10]. *ds-EGFP* was recombined onto *FRT40*, crossed to *hsFlp1; arm-lacZ FRT40* and heatshocked for 1 hr to generate mosaic patches of tissue in which cells expressing Ds-EGFP are juxtaposed to cells expressing Ds. *P[acman]V5-Ft-EGFP* was inserted into an attP site at 96F3 and recombined onto *FRT82* to generate clones by crossing to *hsFlp1; arm-lacZ FRT82.*

The EGUF system [11] was used to generate adult eyes entirely mutant for specific genes. *ey-GAL4* was used to overexpress *UAS-yki* [12] and *UAS-ban* [13] and express RNAi lines against *wts* (KK106174), *hpo* (KK104169) and *ebony* (GD45688) (from VDRC, [14]).

hsFlp1 was used to excise the stop from *Act>stop>fz-EYFP* [15] and *Act>stop>EGFP-dachs* by heatshocking at 48-72hrs for 30 mins to generate small patches of expression. Dachs asymmetry in the absence of Ds and Fj gradients was examined in $ds^{UA071}fj^{d1}$ *Act>stop>EGFP-dachs,* ds^{38K} fj^{P1}*; tubP-GAL4 / UAS-ds* discs.

Histology and Antibodies

Semi-thin sections of adult eyes were made as previously described [16]. Wing and eye discs and S2 cells were fixed in 4% paraformaldehyde and washed in PBS 0.1% Triton-X-100 prior to immunolabelling. Primary antibodies used for histology were rabbit anti-Ds [17], rabbit anti-βGal (Cappel), rabbit anti-GFP (Abcam), mouse anti-Arm (DSHB). A rabbit serum against the intracellular domain of Ft was generated using a His-tagged fusion protein corresponding to amino acids 4665-4859. A rat serum against the N-terminus of Dachs was generated using a His-tagged fusion protein corresponding to amino acids 1-275. Secondary antibodies used were anti-Rb Cy2, RRX and Cy5, anti-mouse Cy5 (Jackson), anti-Rat A568 (Molecular Probes). Actin was labelled with Phalloidin conjugated to A568 (Molecular Probes). Images are averages of confocal sections taken on an Olympus FV1000 confocal and processed in ImageJ and Photoshop.

Quantification of Protein Levels at Junctions

Within experiments confocal images were collected at the same magnification, using the same confocal settings and measurements taken using ImageJ. In the wing disc the PD axis was defined by drawing a line from the hinge through to centre of the wing pouch and oriented in images so this line was a 90º. We defined PD oriented cell boundaries as running perpendicular to this line (0-45º and 135-180º) and AP cell boundaries as parallel (45-135º).

In the eye disc the furrow was taken as the D-V axis and DV and AP cell junctions defined relative to this. To measure Dachs, Ft and Ds antibody staining, a region of 500x500 pixels was drawn close to the pouch/hinge boundary in the dorsal part of the disc. Cell boundaries within this box were marked with a 1 pixel width line and the mean intensity along the line measured. Mean intracellular levels per image were subtracted from junctional measurements. Measurements were segregated into PD (0-45º and 135-180º) and AP (45-135º) oriented boundaries, averaged and the ratio of PD/AP levels calculated per region. 8-10 regions in different discs were measured per genotype and the mean ratio taken. When measuring Dachs/Ds levels around *UAS-ds* overexpression clones, cell boundaries in wildtype tissue parallel to the clone edge were selected and measured up to 6 cells away from the clone. To measure asymmetry of EGFP tagged proteins, GFP staining at junctions on the edges of clones were measured as above. Junctions were catagorised by orientation and mean levels calculated. Data was represented as either mean intensity normalised to distal or equatorial levels or as a ratio of distal/proximal. More than 30 measurements from clones in multiple discs were taken. Throughout the paper one-way ANOVA tests were applied to determine significance levels using either Tukey's test for comparison of all conditions or Dunnett's for comparison between a control and all other conditions.

Analysis of Direction of Fz-EYFP Asymmetry

The direction of Fz-EYFP asymmetry relative to the DV boundaries was measured for individual clones. The position of clones were marked on the wing diagram. Clones that were pointing away from the AP boundary were marked in red.

Cell Culture and Binding Assays

pAct-ft, *pAct-ds*[18] and *pAc5.1-EGFP-dachs* plasmids were transfected into S2 cells singly or in combination, mixed together in binding assays and fixed and immunostained as previously described [18].

Supplemental References

1. Ejsmont, R.K., Sarov, M., Winkler, S., Lipinski, K.A., and Tomancak, P. (2009). A toolkit for high-throughput, cross-species gene engineering in *Drosophila*. Nat. Methods *6*, 435-437.
2. Brand, A.H., and Perrimon, N. (1993). Targeted gene expression as a means of altering cell fates and generating dominant phenotypes. Development *118*, 401-415.
3. Feng, Y., and Irvine, K. (2009). Processing and phosphorylation of the Fat receptor. Proc. Natl. Acad. Sci. USA *106*, 11989-11994.
4. Tweedie, S., Ashburner, M., Falls, K., Leyland, P., McQuilton, P., Marygold, S., Millburn, G., Osumi-Sutherland, D., Schroeder, A., Seal, R., et al. (2009). FlyBase: enhancing Drosophila Gene Ontology annotations. Nucleic Acids Res. *37*, D555-D559.
5. Xu, T., and Rubin, G.M. (1993). Analysis of genetic mosaics in developing and adult *Drosophila* tissues. Development *117*, 1223-1237.
6. Vincent, J.P., Girdham, C.H., and O'Farrell, P.H. (1994). A cell-autonomous, ubiquitous marker for the analysis of *Drosophila* genetic mosaics. Dev. Biol. *164*, 328-331.
7. Strutt, H., Mundy, J., Hofstra, K., and Strutt, D. (2004). Cleavage and secretion is not required for Four-jointed function in *Drosophila* patterning. Development *131*, 881-890.
8. Matakatsu, H., and Blair, S.S. (2004). Interactions between Fat and Dachsous and the regulation of planar cell polarity in the Drosophila wing. Development *131*, 3785-3794.
9. Simon, M.A. (2004). Planar cell polarity in the Drosophila eye is directed by graded Four-jointed and Dachsous expression. Development *131*, 6175-6184.
10. Huang, J., Zhou, W., Watson, A.M., Jan, Y.N., and Hong, Y. (2008). Efficient ends-out gene targeting in Drosophila. Genetics *180*, 703-707.
11. Stowers, R.S., and Schwarz, T.L. (1999). A genetic method for generating Drosophila eyes composed exclusively of mitotic clones of a single genotype. Genetics *152*, 1631-1639.
12. Huang, J., Wu, S., Barrera, J., Matthews, K., and Pan, D. (2005). The Hippo signaling pathway coordinately regulates cell proliferation and apoptosis by inactivating Yorkie, the *Drosophila* Homolog of YAP. Cell *122*, 421-434.
13. Brennecke, J., Hipfner, D.R., Stark, A., Russell, R.B., and Cohen, S.M. (2003). bantam encodes a developmentally regulated microRNA that controls cell proliferation and regulates the proapoptotic gene hid in Drosophila. Cell *113*, 25-36.
14. Dietzl, G., Chen, D., Schnorrer, F., Su, K.C., Barinova, Y., Fellner, M., Gasser, B., Kinsey, K., Oppel, S., Scheiblauer, S., et al. (2007). A genome-wide transgenic RNAi library for conditional gene inactivation in *Drosophila*. Nature *448*, 151-156.
15. Strutt, D.I. (2001). Asymmetric localisation of Frizzled and the establishment of cell polarity in the *Drosophila* wing. Mol. Cell *7*, 367-375.
16. Tomlinson, A., and Ready, D.F. (1987). Cell fate in the *Drosophila* Ommatidium. Dev. Biol. *123*, 264-275.
17. Strutt, H., and Strutt, D. (2002). Nonautonomous planar polarity patterning in *Drosophila*: *dishevelled*-independent functions of *frizzled*. Dev. Cell *3*, 851-863.
18. Brittle, A.L., Repiso, A., Casal, J., Lawrence, P.A., and Strutt, D. (2010). Four-Jointed Modulates Growth and Planar Polarity by Reducing the Affinity of Dachsous for Fat. Curr. Biol. *20*, 803-810.
